# Mealybug species from Chilean agricultural landscapes and main factors influencing the genetic structure of *Pseudococcus viburni*

**DOI:** 10.1038/srep16483

**Published:** 2015-11-12

**Authors:** Margarita C. G. Correa, Eric Lombaert, Thibaut Malausa, Didier Crochard, Andrés Alvear, Tania Zaviezo, Ferran Palero

**Affiliations:** 1 Facultad de Agronomía e Ingeniería Forestal, Pontificia Universidad Católica de Chile, Casilla 306-22, Santiago, Chile; 2 INRA, Univ. Nice Sophia Antipolis, CNRS, UMR 1355-7254 Institut Sophia Agrobiotech, 06900 Sophia Antipolis, France; 3 Xilema Control Biológico, Ruta 60, San Pedro, Quillota, Chile

## Abstract

The present study aimed to characterize the distribution of mealybug species along Chilean agro-ecosystems and to determine the relative impact of host plant, management strategy, geography and micro-environment on shaping the distribution and genetic structure of the obscure mealybug *Pseudococcus viburni*. An extensive survey was completed using DNA barcoding methods to identify Chilean mealybugs to the species level. Moreover, a fine-scale study of *Ps. viburni* genetic diversity and population structure was carried out, genotyping 529 *Ps. viburni* individuals with 21 microsatellite markers. Samples from 16 localities were analyzed using Bayesian and spatially-explicit methods and the genetic dataset was confronted to host-plant, management and environmental data. Chilean crops were found to be infested by *Ps. viburni, Pseudococcus meridionalis, Pseudococcus longispinus* and *Planococcus citri*, with *Ps. viburni* and *Ps. meridionalis* showing contrasting distribution and host-plant preference patterns. *Ps. viburni* samples presented low genetic diversity levels but high genetic differentiation. While no significant genetic variance could be assigned to host-plant or management strategy, climate and geography were found to correlate significantly with genetic differentiation levels. The genetic characterization of *Ps. viburni* within Chile will contribute to future studies tracing back the origin and improving the management of this worldwide invader.

Scale insects are worldwide-distributed agricultural pests that cause major economic losses of billions of dollars every year^1^,^2^, either through direct impact on crops or quarantine export restrictions^3^,^4^. Commonly known as mealybugs, scale insects belonging to the family Pseudococcidae are particularly difficult to manage^5^,^6^. Their small size and cryptic habits render them very inconspicuous so that they easily escape phytosanitary inspections. For example, the obscure mealybug *Pseudococcus viburni* (Signoret) has expanded across the globe and is now present in over 60 countries^7^. Chemical control of pseudococcids is often ineffective due to their concealed nature and patchy distributions^4^,^8^. As a consequence, many management programs rely on classical biological control and especially the use of encyrtid parasitoids^6^,^9^. The response to particular biological control agents varies depending on the mealybug species^10^, so characterizing the diversity and distribution of mealybugs becomes important for both fundamental research as well as in applied pest management.

The spatial distribution of agro-ecosystems is a key factor determining the species composition and population genetic structure of agricultural pests^11^,^12^. Chilean highly structured agricultural landscape represents an excellent case study, with 83% of fruit orchards being found in a 700 km long stretch of land (between Coquimbo and Maule regions) and 20 fruit species concentrating about 90% of the cultivated area^13^. Geographical and environmental features of the country also contribute to further structuring Chilean agro-ecosystems. In the Norte Chico area (26° S to 32° S), which includes the administrative regions of Atacama (partly overlapping the Atacama Desert) and Coquimbo, there are semi-desert conditions with extreme climate variations. In the Central Chile area (32° S to 37° S), comprising the administrative regions of Valparaíso, Metropolitana, O’Higgins, Maule and the Northern part of Biobío, climate is mostly Mediterranean, with reliable alternation of warm, dry summers and cooler, rainier winter seasons^14^. The Norte Chico climate is mostly favorable to vineyards, while the Central Chile area includes both vineyards and fruit orchards (e.g. orange trees, apple trees, plum trees and pears). By running parallel to the Ocean, the coastal mountains and Andes cordillera further enclose vineyards and fruit orchards in isolated valleys, even within administrative regions.

National production of grapes and fruit orchards in Chile has significantly increased during the last decade^15^. Mealybug detection after phytosanitary inspections is responsible for over 45% fruit rejections in exports, and pest infestations must be controlled for the sustainability of the Chilean agro-industry^16^. Correct identification is essential for pest management, however previous reports on mealybugs have evidenced considerable difficulties when using morphology-based methods^17^,^18^,^19^. In a preliminary study using a DNA barcoding approach, Correa *et al.*^20^ found *Pseudococcus viburni* (Signoret) to be the most common mealybug species in Chilean vineyards, followed by *Pseudococcus meridionalis* Prado and *Pseudococcus cribata* González. In contrast with previous studies though, no *Pseudococcus longispinus* (Targioni Tozzetti) or *Planococcus ficus* (Signoret) were found, which could result from the limited area being covered (two regions) and the fact that only vineyards were sampled^20^.

The present study aimed at characterizing the diversity and distribution of mealybug species infesting Chilean agro-ecosystems, including both vineyards and deciduous fruit crops. In order to achieve this goal, we completed an extensive survey between Atacama and the Northern part of Biobío (covering most cultivated areas in Chile) and using DNA barcoding methods to identify mealybug samples to the species level. Moreover, a fine-scale study of *Ps. viburni* genetic diversity and population connectivity was carried out using microsatellite markers. A comprehensive analysis of *Ps. viburni* samples allowed us to determine the relative impact of host plant, management strategy, geography and micro-environment on shaping the distribution and genetic structure of the pest.

## Methods

### Sampling and molecular identification

A total of 38 sampling sites were surveyed along Chile in 2012 and 2013 (Table 1). Sample collection was performed by taking one mealybug specimen per plant (20 – 49 mealybugs per site) to avoid collecting individuals from a single family. Specimens were stored in absolute ethanol at −20 °C until DNA extraction. Given that mealybug species are difficult to distinguish based on their morphology, DNA barcoding identification was performed. Genomic DNA was extracted from at least five individuals per locality using the DNeasy extraction kit (QIAGEN, Hilden, Germany), and the conserved 28S ribosomal marker was PCR-amplified using the primers S3690 (5′-GAGAGTTMAASAGTACGTGAAAC-3′) and A4394 (5′-TCGGARGGAACCAGC-TACTA-3′)^21^. PCR reactions were performed in a total reaction volume of 25 μL and following the protocol of Abd-Rabou *et al.*^22^. The PCR mix was composed of 12.50 μL 2X Tmix (QIAGEN, Hilden, Germany), 0.125 μL of each primer (initial concentration of 100 μM) and 10.25 μL of ultrapure water. PCR conditions were: initial denaturation at 95°C for 15 min; 35 cycles of denaturation at 95 °C for 30 s, hybridization at 58 °C for 90 s, elongation for 60 s; final extension at 72 °C for 10 min. PCR products were then analyzed on a QIAxcel Advanced System (QIAGEN) and sent for sequencing to Genoscreen (Lille, France) or Beckman Genomics (Takeley, United Kingdom). PCR products were sequenced on both strands and consensus sequences and alignments were created manually with Bioedit version 7.01^23^.

### Microsatellite Diversity Analyses

Out of the initial 38 sampling sites, a total of 16 samples were identified as *Ps. viburni* (see results section) and kept for further analysis using the 21 polymorphic microsatellite markers developed by Correa *et al.*^24^. Genotyping was carried out in a total of 529 individuals from those *Ps. viburni* populations. Individual PCRs were performed in a total volume of 10 μl, with cycling conditions as in Correa *et al.*^24^. PCR products were separated by electrophoresis using an ABI 3700 sequencer (Applied Biosystems) and 500 LIZ GeneScan^TM^ size standard. Allelic profiles were obtained for each individual using the Genemarker^TM^ v1.75 software (SoftGenetics LLC).

For each sampling locality, mean number of alleles, observed (H_O_) and expected (H_E_) heterozygosity were computed using the software GeneClass v2^25^. Allelic richness was estimated standardizing sample size with the R package standArich (available online at http://www.ccmar.ualg.pt/maree/software.php?soft=sarich). Deviations from Hardy-Weinberg Equilibrium (HWE) were tested for each locus and sampling locality using the software Genepop v4.2^26^. Per locus p-values were corrected for multiple testing within each population using the false discovery rate (FDR) procedure^27^. The presence of null alleles was evaluated with the FreeNA software, and 10,000 bootstrap replicates were used to test for significance^28^. Genetic signals of recent bottlenecks affecting *Ps. viburni* were analyzed using the software BOTTLENECK v.1.2.02^29^.

### Genetic connectivity of *Pseudococcus viburni* populations

Pairwise *F*ST estimates^30^ and Fisher’s exact probability tests for genotypic differentiation^31^ were performed using the software Genepop v4.2^26^. Levels of genetic differentiation between sample sites were also estimated using the *D*ST statistic, which includes a multiplicative partitioning of diversity, based on the effective number of alleles rather than on the expected heterozygosity^32^. Pairwise *D*ST statistic estimates and their significance using 1,000 bootstrap replicates were obtained with the pair.pops.Dest function in the DEMETICS package in R (http://www.r-project.org/). Sampling localities were grouped with the neighbor-joining algorithm and the pairwise genetic chord distance^33^ as implemented in the software Populations v1.2.30 (http://bioinformatics.org/populations/).

Genetic clusters were then inferred by minimizing deviation from HWE and linkage disequilibrium (LD) with the Bayesian clustering approach implemented in STRUCTURE^34^. Parameters were estimated assuming an admixture model with correlated allele frequencies and no prior location information. We carried out 20 replicate runs for each value of the number of clusters (K), set between 1 and 16 (i.e. the number of sampling sites). Each run consisted of a burn-in period of 200,000 Markov chain Monte Carlo (MCMC) iterations, followed by 1,000,000 MCMC iterations. The highest level of genetic structure was inferred through the ΔK approach as implemented in STRUCTURE Harvester^35^,^36^. Finally, a discriminant analysis of principal components (DAPC) was used to identify clusters of genetically-related genotypes. DAPC (like the sPCA method presented below) differs from the Bayesian method in STRUCTURE by not assuming HWE or linkage equilibrium^37^.

### Spatial genetic structure analyses

Isolation by distance (IBD) was tested by performing a linear regression of the genetic distances (FST or DST) and the geographical distances (Km) and obtaining the Pearson’s correlation coefficient. Matrices of genetic distance and geographical distance were subjected to a Mantel test with 10,000 permutations in Arlequin v.3.5^38^. A spatially-explicit multivariate method (sPCA: spatial analysis of principal components) was used to further explore the spatial patterns of genetic variability between sites^39^. Spatial connectivity networks were built using the sampling location data and three common algorithms (Delaunay triangulation, relative neighbor and sphere of influence)^40^. In order to select for the network that better fits the genetic data, the corrected Akaike Information Criteria (AICc) was computed for each graph using the ortho.AIC function of the spacemakeR package^41^. The connectivity graph with the highest AICc was used to test for the presence of spatial autocorrelation against the null hypothesis that allele frequencies are distributed at random. Spatial correlation can be either positive or negative, resulting in principal components with positive or negative eigenvalues. Principal components with significant positive eigenvalues indicate that sites are genetically more similar to their neighbors than expected by chance (global structures), while those with significant negative eigenvalues highlight local differentiation among neighboring sites^39^. Spatial autocorrelation (Moran’s I) was tested for each component using the permutation procedure implemented in the spdep package and 1,000 permutations^41^.

### Non-genetic factors influencing population structure

Several analyses of molecular variance (AMOVA) were performed in order to evaluate the importance of non-genetic categorical factors on the distribution of genetic diversity. In particular, sample sites were grouped according to (i) administrative region (AT, CQ, VL, MT, OH or ML), (ii) type of agricultural management (conventional or organic production), and (iii) host plant (grape, apple, plum or pear). The significance for the AMOVAs was obtained through non-parametric permutation procedures (with 20,000 permutations) as implemented in Arlequin v3.5^38^. To examine whether non-genetic environmental factors may influence genetic differentiation levels in *Ps. viburni*, temperature and precipitation variables were recovered to 1-km spatial resolution from WorldClim version 1.4 (http://www.worldclim.org)^42^. The climate variables included temperature and precipitation parameters, namely: i) annual mean temperature, ii) maximum temperature of the warmest month, iii) minimum temperature of the coldest month, iv) annual precipitation and v) precipitation seasonality (Coefficient of Variation). A Spearman’s rank correlation (ρ) test was then carried out between pairwise genetic distances (FST or DST) and the Euclidean distance between climate variables for each pair of sampling sites. Spearman’s correlation measures association based on the rank differences between two vectors and indicates how well a monotonic function describes their relationship.

## Results

### Molecular identification of mealybug species

**Figure 1:**
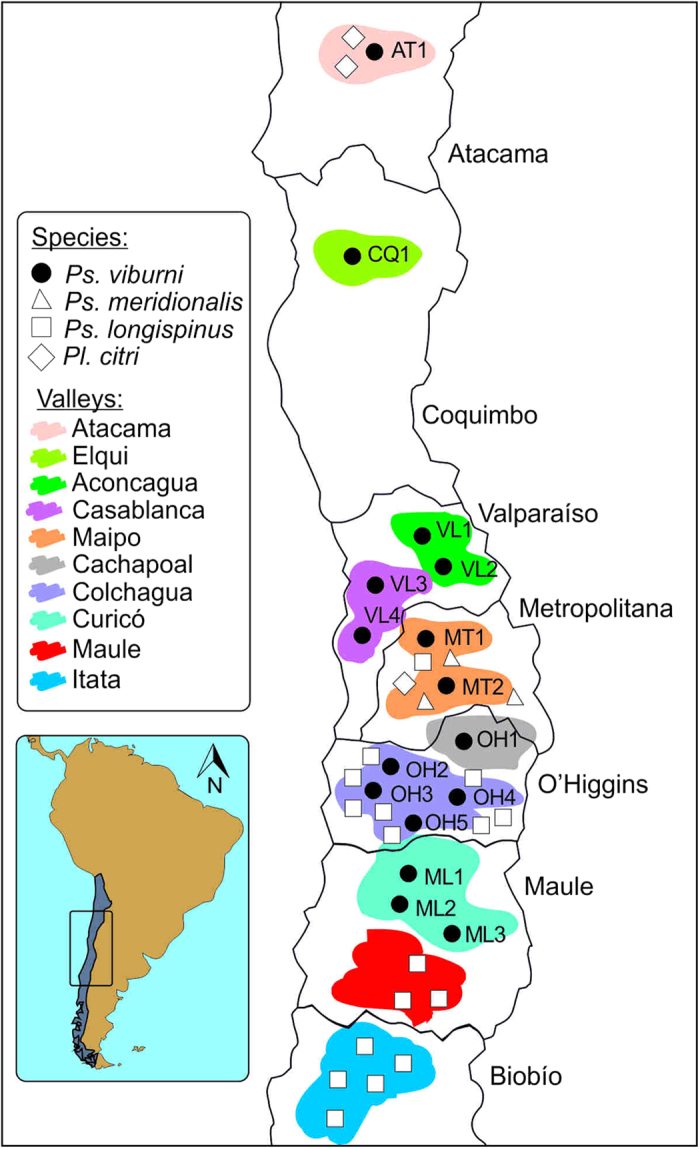
Spatial distribution of mealybug species found in Chilean valleys. Sampling localities are indicated using different shapes depending on the species found. Limits between administrative regions are drawn as black lines and valleys are highlighted using different colors. Figure created using the software CorelDRAW X6.

The present survey of scale insects infesting Chilean crops identified four mealybug species (*Planococcus citri, Pseudococcus longispinus, Pseudococcus meridionalis* and *Pseudococcus viburni*). *Ps. viburni* was the most widespread pest, being found on vineyards, apple trees, plum trees or pear trees in six out of seven administrative regions sampled: Atacama (AT), Coquimbo (CQ), Valparaíso (VL), Metropolitana (MT), O’Higgins (OH) and Maule (ML) (Table 1; Fig. 1). *Ps. longispinus* was also present in several regions and dominated apple fields south of Maule and in Biobío. Finally, *Pl. citri* and *Ps. meridionalis* were only found sporadically on vineyards or orange trees (Fig. 1).

### Microsatellite Diversity Analyses

*Ps. viburni* samples showed an overall mean number of alleles of 3.06, with values ranging from 2.29 (ML1) to 3.62 (VL3). The overall mean allelic richness was 2.97 (N = 20 genotypes), with MT2 presenting the highest (3.52) and AT1 the lowest (2.20) values (Table 2). After correction by the FDR procedure, seven sites still showed significant HWE deviations (Table 2). Frequency of null alleles for each microsatellite marker ranged from 0.002 (PV071) to 0.133 (PV060). Nevertheless, the presence of null alleles did not affect global FST estimates, and similar values were obtained using the raw dataset (FST = 0.126) or the corrected dataset (FST = 0.125). Similarly, pairwise FST values did not vary after FREENA correction for the presence of null alleles.

### Genetic connectivity of Pseudococcus viburni populations

**Figure 2:**
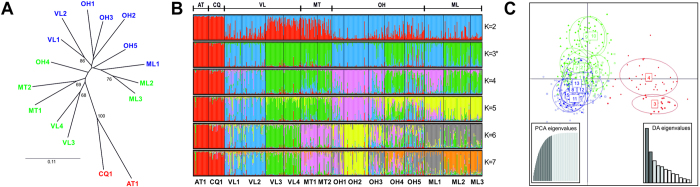
Genetic clustering of *Ps. viburni* populations from Chile. Results obtained using (**A**) Distance-based neighbor-joining tree, with node support obtained using 1,000 bootstrap replicates over loci (cut-off value >65); (**B**) Bayesian clustering method implemented in STRUCTURE; (**C**) Discriminant analysis of principal components (DAPC). Populations have been color-coded in figures 2A and 2C following the clusters obtained from STRUCTURE.

Pairwise FST values ranged from 0.011 (between ML2 and ML3) to 0.350 (between AT1 and ML3) (Table 3). All pairs of *Ps. viburni* samples showed significant differentiation levels after Fisher’s exact tests (P < 0.05). The results obtained using the distance-based neighbor-joining approach showed a clear support for the separation of AT1 and CQ1 from the remaining samples (Fig. 2a). Despite most bootstrap values were low, a strong support was found for some groups sites, such as VL1-VL2, ML2-ML3, MT1-MT2 and VL3-VL4. Structure Harvester selected K = 3 as the best representative number of clusters based on likelihood and ΔK (Ln = −16457; ΔK = 443.35). The first cluster included the two Northernmost sites (AT1 and CQ1), a second cluster was composed of samples from the Aconcagua (VL1 and VL2), Cachapoal (OH1), Colchagua (OH2 and OH3) and Curicó (ML1) valleys; and a third cluster was formed by the Casablanca (VL3 and VL4), Maipo (MT1 and MT2), Colchagua (OH5) and Curicó (ML2 and ML3) valleys (Figs 1, 2b). Finally, the DAPC method also support the presence of a cluster including AT1 and CQ1 samples, but indicated large overlapping between the samples assigned to the second (blue) and third (green) clusters given by STRUCTURE (Fig. 2c).

### Spatial genetic structure analyses

AMOVA results showed that a significant 7.73% of the total genetic variability can be explained by administrative regions (FCT = 0.074; P < 0.0001). A significant positive correlation was found between geographical distance and either FST (r^2^ = 0.79; P < 0.0001) or DST values (r^2^ = 0.75; P < 0.0001) when considering all the 16 sample sites, indicating the presence of IBD. When the IBD test was performed without the two most distant localities (AT1 and CQ1), a weaker but still significant pattern was also observed both using FST (r^2^ = 0.068; P = 0.028) or DST values (r^2^ = 0.121; P = 0.004).

**Figure 3:**
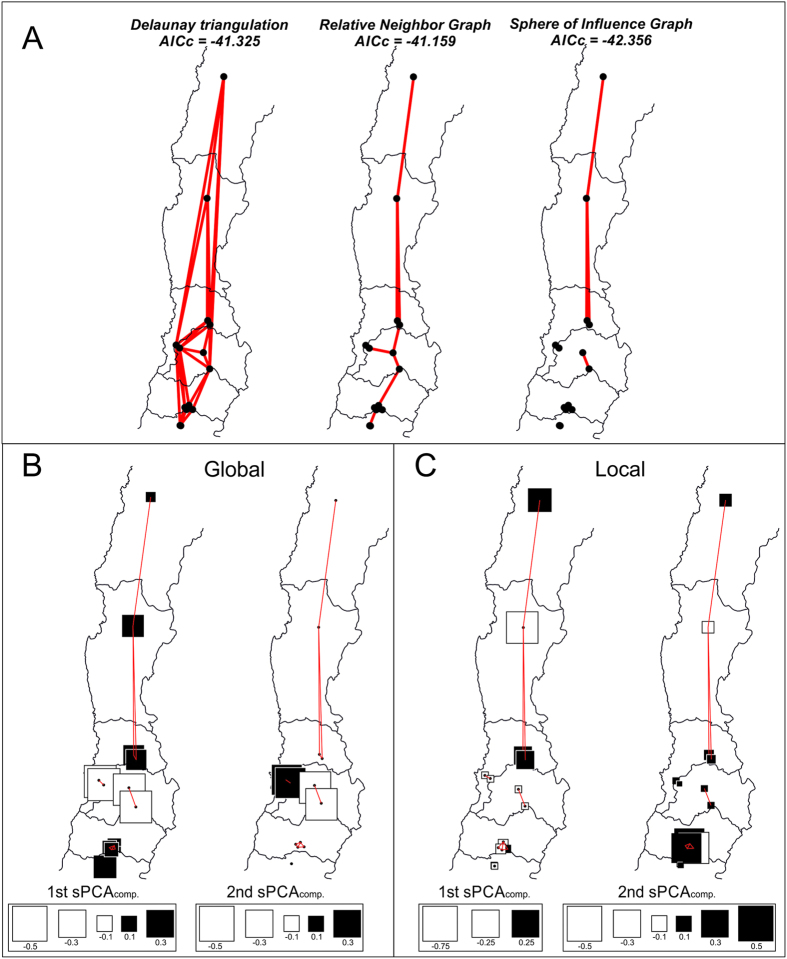
Spatial genetic structure of *Ps. viburni* populations from Chile. (**A**) Different connectivity networks tested; (**B**) The two main global components of the sPCA analysis, showing that the populations from the Valparaiso (VL3-VL4) and Metropolitana (MT1-MT2) regions have larger negative scores (white squares) than locations on the other clusters; (**C**) Plot of the two components with the largest negative values, showing that local changes are significant in the northern samples and within the O’Higgins area (both white and black squares present within clusters). Figure created using the software CorelDRAW X6.

According to the corrected AIC criterion, the best connectivity graph for explaining the *Ps. viburni* allele frequency data was obtained from the Sphere of Influence (SoI) algorithm (AICc = −42.356; non-zero links = 34), which showed a lower AICc value than both the Delaunay triangulation (AICc = −41.325; non-zero links = 78) or the Relative Neighbor Graph (AICc = −41.159; non-zero links = 30) (Fig. 3A). The SoI algorithm differs from the Relative Neighbor algorithm by creating a disconnected graph despite having a larger number of nonzero links. The graphical representation of the scores of the first and second global components (with significant positive eigenvalues) showed that samples from the Valparaiso (VL3-VL4) and Metropolitana (MT1-MT2) regions have different allele composition than other clusters (negative scores shown as large white squares in Fig. 3B). A graphical representation of the components with significantly negative eigenvalues shows that local changes can be observed between the northernmost localities and within the O’Higgins area (both white and black squares present within connected sites in Fig. 3C).

### Non-spatial factors influencing population structure

No significant genetic variance could be assigned to differences among organic and conventional management strategies (FCT = −0.0006; p-value = 0.402) or among host plants (FCT = −0.015; p-value = 0.587) (Table 4). As for the effect of climatic environmental factors, Spearman’s rank test indicated a strong correlation between genetic distances and differences in maximum temperature of the warmest month (ρ = 0.615; p-value <0.001), minimum temperature of the coldest month (ρ = 0.645; p-value <0.001), annual precipitation (ρ = 0.523; p-value <0.001) and precipitation seasonality (ρ = 0.537; p-value <0.001). However, the correlation between genetic distances and annual mean temperature was much smaller and non-significant after FDR correction (ρ = 0.239; p-value = 0.008).

## Discussion

The distribution of insect pest species across agricultural ecosystems can be shaped by multiple factors, ranging from specific associations with the cultivated plant, environmental gradients or the use of pesticides. The study of these multiple factors, together with an accurate discrimination between mealybug species, are determinant for an adequate adjustment of management strategies. Previous morphology-based surveys of Chilean mealybugs have found *Ps. viburni, Ps. meridionalis, Ps. cribata, Ps. longispinus* and *Pl. ficus*, but the only molecular study performed up to date could not recover the last two species^20^. Our results establish, thanks to DNA barcoding methods, the presence of both *Ps. longispinus* and *Pl. citri* infesting Chilean crops. Therefore, previous reports of *Pl. ficus* could correspond in fact to *Pl. citri*, given the difficulties of morphology-based identification of mealybugs. The main *Pseudococcus* species infesting Chilean fruit crops was the obscure mealybug *Ps. viburni*, followed by *Ps. longispinus* and *Ps. meridionalis*.

Both *Ps. viburni* and *Ps. longispinus* were recovered from vineyards and fruit orchards. Nevertheless, while *Ps. viburni* occurred mainly in vineyards around the central regions of Chile (Valparaíso and Metropolitana) and became less frequent south of O’Higgins, *Ps. longispinus* showed an opposite pattern and was more abundant in Apple orchards south of that region. These different spatial distribution and host-plant preference patterns are in agreement with previous studies on the biology and systematics of Pseudococcidae^4^. Indeed, *Ps. viburni* belongs to the grape mealybug (*Ps. maritimus*) species complex, while the long-tailed mealybug (*Ps. longispinus*) and related species are commonly found on fruit orchards (e.g. avocado^43^,^44^ and pear^45^).

The host preference observed for different species did not extend to different populations within *Ps. viburni*, since no significant genetic variance could be assigned to host plant or management strategy. This lack of genetic structuring by host indicates that *Ps. viburni* populations are able to infest fruit orchards in an opportunistic way despite their preference for vines^4^. Rather than host plant or management strategy, our results highlight the importance of geography and environmental factors in shaping the intra-species population genetic structure of the obscure mealybug. The integrative analyses of micro-climatic variables and genetic markers show that the severity of climate (i.e. differences in the extremes of temperature or precipitation seasonality) is significantly correlated with genetic differentiation levels. Meteorological changes or edaphic factors have been previously proposed to modify the physiology of the plant and alter its resistance to scale insect attack^46^,^47^, but this is the first study to provide evidence on the importance of environmental factors on shaping scale insects distribution and genetic structure.

Climate differences might correlate with geographical distances, so that spurious genetic-climate correlations could result from an isolation by distance pattern. AMOVA, IBD and sPCA analyses all support the presence of reduced connectivity between *Ps. viburni* samples related to their spatial distribution. The fact that IBD remained significant even after excluding the two most distant populations indicates that genetic differentiation is also important at a local scale, where climatic differences are minimal. Therefore, geography cannot be discarded as a key factor shaping the population genetic structure of the obscure mealybug. In a recent study, the maritime pine bast scale (*Matsucoccus feytaudi*) was found to be highly structured geographically, and the authors relate this fact to the limited dispersal capacity of the insect and the patchy distribution of the obligate host^48^. Mealybug species are known to present low dispersal abilities, so long distance movements could only be driven by human activities and agricultural practices^4^. The peculiar shape of Chile, being only 177 km wide in average but 4300 km long, together with the low mobility of mealybugs, could well explain the observed geography-driven genetic structure of *P. viburni*.

The identification of well-differentiated groups within *Ps. viburni* is an important contribution to design effective control strategies for pest management both in Chile and elsewhere. Even though the exact center of origin for the obscure mealybug remains unclear, South America has been suggested as the most likely native area. Therefore, the genetic characterization of *Ps. viburni* within Chile will contribute to future studies tracing back the origin and improving the management of this worldwide invader (e.g. allowing to search for specific parasitoids within the source populations).

## Additional Information

**How to cite this article**: Correa, M. C. G. *et al.* Mealybug species from Chilean agricultural landscapes and main factors influencing the genetic structure of *Pseudococcus viburni. Sci. Rep.* **5**, 16483; doi: 10.1038/srep16483 (2015).

## Figures and Tables

**Table 1:** Mealybug populations sampled along Chile and molecular identification using the 28S gene region.

Pop	Locality	Region	Host	Collector	Species identification
AT1	Copiapó	Atacama	Grape	M. Correa	*Pseudococcus viburni*
AT2	Copiapó	Atacama	Grape	M. Correa	*Planococcus citri*
AT3	Copiapó	Atacama	Grape	M. Correa	*Planococcus citri*
CQ1	Vicuña	Coquimbo	Grape	M. Correa	*Pseudococcus viburni*
VL1	San Felipe	Valparaíso	Grape	M. Correa	*Pseudococcus viburni*
VL2	Los Andes	Valparaíso	Grape	M. Correa	*Pseudococcus viburni*
VL3	Casablanca	Valparaíso	Grape	A. Galaz/L. Segovia	*Pseudococcus viburni*
VL4	Casablanca	Valparaíso	Grape	A. Galaz/L. Segovia	*Pseudococcus viburni*
MT1	Pudahuel	Metropolitana	Grape	A. Galaz/L. Segovia	*Pseudococcus viburni*
MT2	Paine	Metropolitana	Plum	A. Galaz/L. Segovia	*Pseudococcus viburni*
MT3	Pirque	Metropolitana	Grape	M. Correa	*Pseudococcus meridionalis*
MT4	Los Morros	Metropolitana	Grape	A. Galaz/L. Segovia	*Pseudococcus longispinus, Pseudococcus meridionalis*
MT5	Linderos	Metropolitana	Grape	A. Galaz/L. Segovia	*Pseudococcus meridionalis,*
MT6	María Pinto	Metropolitana	Orange	M. Correa	*Planococcus citri*
OH1	Placilla	O’Higgins	Apple	A. Romero	*Pseudococcus viburni*
OH2	Nancagua	O’Higgins	Grape	M. Correa	*Pseudococcus viburni*
OH3	Nancagua	O’Higgins	Grape	M. Correa	*Pseudococcus viburni*
OH4	Chimbarongo	O’Higgins	Grape	M. Correa	*Pseudococcus viburni*
OH5	Chépica	O’Higgins	Grape	M. Correa	*Pseudococcus viburni*
OH6	Peumo	O’Higgins	Pear	K. Buzzetti	*Pseudococcus longispinus*
OH7	Peumo	O’Higgins	Grape	K. Buzzetti	*Pseudococcus longispinus*
OH8	Peumo	O’Higgins	Grape	K. Buzzetti	*Pseudococcus longispinus*
OH9	Peumo	O’Higgins	Apple	K. Buzzetti	*Pseudococcus longispinus*
OH10	Las Cabras	O’Higgins	Grape	K. Buzzetti	*Pseudococcus longispinus*
OH11	Doñihue	O’Higgins	Apple	K. Buzzetti	*Pseudococcus longispinus*
OH12	Pichidegua	O’Higgins	Orange	M. Correa	*Pseudococcus longispinus,*
OH13	Las Cabras	O’Higgins	Orange	M. Correa	*Pseudococcus longispinus*
ML1	Molina	Maule	Apple	M. Correa	*Pseudococcus viburni*
ML2	Molina	Maule	Grape	M. Correa	*Pseudococcus viburni*
ML3	Molina	Maule	Pear	M. Correa	*Pseudococcus viburni*
ML4	Molina	Maule	Apple	K. Buzzetti	*Pseudococcus longispinus*
ML5	Molina	Maule	Apple	K. Buzzetti	*Pseudococcus longispinus*
ML6	Molina	Maule	Apple	K. Buzzetti	*Pseudococcus longispinus*
BB1	Angol	Bio Bio	Apple	K. Buzzetti	*Pseudococcus longispinus*
BB2	Angol	Bio Bio	Apple	K. Buzzetti	*Pseudococcus longispinus*
BB3	Angol	Bio Bio	Apple	K. Buzzetti	*Pseudococcus longispinus*
BB4	Angol	Bio Bio	Apple	K. Buzzetti	*Pseudococcus longispinus*
BB5	Angol	Bio Bio	Apple	K. Buzzetti	*Pseudococcus longispinus*

**Table 2:** Genetic diversity and categorical data of *Pseudococcus viburni* samples. Microsatellite data: mean number of alleles (Na), allelic richness (Â) for n=20 genotypes, observed (Ho) and expected (He) heterozygosity with standard deviation (s.d.), and inbreeding coefficients (FIS).

Pop	Locality	Region	Host	Management Strategy	Microsatellite Data									FIS
					N	Na	Â	Ho		s.d	He		s.d	
AT1	Copiapó	Atacama	Grape	Traditional	28	2.33	2.2	0.270	±	0.324	0.255	±	0.263	0,101
CQ1	Vicuña	Coquimbo	Grape	Traditional	29	2.71	2.64	0.331	±	0.264	0.342	±	0.256	0,038
VL1	San Felipe	Valparaíso	Grape	Traditional	32	3.38	3.19	0.330	±	0.217	0.380	±	0.253	0,100
VL2	Los Andes	Valparaíso	Grape	Traditional	30	3.52	3.18	0.397	±	0.248	0.414	±	0.243	0,049
VL3	Casablanca	Valparaíso	Grape	Organic	33	3.62	3.43	0.435	±	0.238	0.438	±	0.218	−0,001
VL4	Casablanca	Valparaíso	Grape	Organic	32	3.38	3.33	0.355	±	0.251	0.390	±	0.234	0,094
MT1	Pudahuel	Metropolitana	Grape	Organic	27	3.48	3.50	0.390	±	0.231	0.424	±	0.227	0,088
MT2	Paine	Metropolitana	Plum	Organic	43	3.19	3.52	0.373	±	0.261	0.410	±	0.215	0,110
OH1	Placilla	O’Higgins	Apple	Organic	22	2.91	2.90	0.395	±	0.273	0.391	±	0.234	0,041
OH2	Nancagua	O’Higgins	Grape	Organic	37	2.62	2.28	0.331	±	0.251	0.311	±	0.232	−0,048
OH3	Nancagua	O’Higgins	Grape	Organic	45	3.14	3.15	0.367	±	0.210	0.410	±	0.208	0,097
OH4	Chimbarongo	O’Higgins	Grape	Organic	36	3.38	3.17	0.379	±	0.212	0.426	±	0.235	0,110
OH5	Chépica	O’Higgins	Grape	Traditional	30	2.95	2.84	0.324	±	0.196	0.369	±	0.198	0,133
ML1	Molina	Maule	Apple	Organic	35	2.29	2.23	0.295	±	0.229	0.295	±	0.218	0,027
ML2	Molina	Maule	Grape	Traditional	49	3.43	2.96	0.344	±	0.223	0.363	±	0.221	0,066
ML3	Molina	Maule	Pear	Traditional	21	2.57	2.96	0.323	±	0.229	0.327	±	0.220	0,022

**Table 3:** Pairwise FST (lower matrix) and DST values (upper matrix) observed between *Pseudococcus viburni* samples from Chile. All Fisher’s exact tests for population differentiation were significant at the 0.05 threshold.

	AT1	CQ1	VL1	VL2	VL3	VL4	MT1	MT2	OH1	OH2	OH3	OH4	OH5	ML1	ML2	ML3
AT1	–	0.1243	0.2218	0.2143	0.2032	0.1902	0.2175	0.2237	0.2249	0.2271	0.2046	0.2096	0.1727	0.2244	0.2541	0.2423
CQ1	0.1701	–	0.1908	0.2060	0.1497	0.1664	0.1589	0.1509	0.1833	0.2262	0.1845	0.1920	0.1490	0.2274	0.2229	0.2426
VL1	0.2682	0.2212	–	0.0350	0.1041	0.1033	0.0761	0.0980	0.0541	0.0671	0.0369	0.0585	0.0490	0.0812	0.0610	0.0612
VL2	0.2959	0.2225	0.0314	–	0.1031	0.1035	0.0893	0.1061	0.0925	0.0891	0.0620	0.0812	0.0754	0.0906	0.0926	0.0846
VL3	0.2534	0.1734	0.0773	0.0522	–	0.0344	0.1119	0.0885	0.1369	0.1614	0.1085	0.0718	0.0795	0.1308	0.0800	0.0909
VL4	0.3408	0.2642	0.0959	0.0702	0.0742	–	0.1147	0.0732	0.1364	0.1490	0.1021	0.0709	0.0800	0.1126	0.0821	0.0880
MT1	0.2337	0.1493	0.0920	0.0956	0.0798	0.0767	–	0.0609	0.0814	0.1391	0.0717	0.0763	0.0852	0.1360	0.1115	0.1033
MT2	0.2335	0.1622	0.0937	0.0946	0.0803	0.0801	0.0303	–	0.0729	0.1266	0.0646	0.0628	0.0792	0.0958	0.0875	0.0805
OH1	0.2660	0.1822	0.0820	0.0767	0.0822	0.1087	0.1012	0.1017	–	0.0745	0.0248	0.0457	0.0677	0.0841	0.1003	0.0979
OH2	0.3402	0.2878	0.1094	0.0928	0.1231	0.1331	0.1651	0.1599	0.1555	–	0.0521	0.0761	0.0912	0.0795	0.0974	0.0859
OH3	0.2723	0.2081	0.0649	0.0437	0.0383	0.0806	0.0975	0.0900	0.0721	0.0727	–	0.0348	0.0347	0.0428	0.0688	0.0554
OH4	0.2582	0.1991	0.0700	0.0569	0.0485	0.0323	0.0596	0.0593	0.0719	0.0861	0.0392	–	0.0453	0.0575	0.0372	0.0409
OH5	0.2952	0.1950	0.1052	0.1018	0.0898	0.0942	0.0858	0.0639	0.0641	0.1562	0.0642	0.0580	–	0.0589	0.0584	0.0552
ML1	0.3077	0.2214	0.1018	0.0695	0.0778	0.1147	0.1327	0.1375	0.0868	0.0976	0.0334	0.0454	0.0775	–	0.0457	0.0371
ML2	0.3516	0.2928	0.1120	0.1076	0.0917	0.0719	0.1433	0.1223	0.1448	0.1325	0.0587	0.0697	0.1073	0.1142	–	0.0142
ML3	0.3538	0.2838	0.0778	0.0597	0.0606	0.0018	0.0720	0.0748	0.0948	0.1118	0.0541	0.0227	0.0936	0.1092	0.0519	–

**Table 4:** Global Analyses of Molecular Variance (AMOVA) as a weighted average over loci carried out to compare the effect of categorical factors on the genetic structure of *Pseudococcus viburni*.

	Source of Variation	df	SS	Variance components	% variation	F statistics
Administrative Regions	Among groups	5	350.639	0.305	7.37	F_CT_ = 0.074
	Among populations within groups	10	210.896	0.263	6.36	F_SC_ = 0.069
	Within populations	1042	3717.699	3.568	86.28	
					
Management Strategy	Among groups	1	38.407	−0.002	−0.06	F_**CT**_ = −0.001
	Among populations within groups	14	523.128	0.515	12.63	**F_SC_ = 0.126**
	Within populations	1042	3717.699	3.568	87.43	
					
Host Plant	Among groups	3	75.003	−0.058	−1.44	F_**CT**_ = −0.015
	Among populations within groups	12	486.531	0.535	13.23	**F_SC_ = 0.131**
	Within populations	1042	3717.699	3.568	88.21	

Significant results (p < 0.05) after 20,000 permutations are indicated in bold.
